# The rate of protein synthesis in hematopoietic stem cells is limited partly by 4E-BPs

**DOI:** 10.1101/gad.282756.116

**Published:** 2016-08-01

**Authors:** Robert A.J. Signer, Le Qi, Zhiyu Zhao, David Thompson, Alla A. Sigova, Zi Peng Fan, George N. DeMartino, Richard A. Young, Nahum Sonenberg, Sean J. Morrison

**Affiliations:** 1 Howard Hughes Medical Institute, Children's Research Institute, Department of Pediatrics, University of Texas Southwestern Medical Center, Dallas, Texas 75390, USA;; 2 Division of Regenerative Medicine, Department of Medicine, Moores Cancer Center, University of California at San Diego, La Jolla, California 92093, USA;; 3 Department of Physiology, University of Texas Southwestern Medical Center, Dallas, Texas 75390, USA;; 4 Whitehead Institute for Biomedical Research, Massachusetts Institute of Technology, Cambridge, Massachusetts 02142, USA;; 5 Department of Biology, Massachusetts Institute of Technology, Cambridge, Massachusetts 02139, USA;; 6 Department of Biochemistry, Goodman Cancer Centre, McGill University, Montreal, Quebec H3A 1A3, Canada

**Keywords:** 4E-BP, protein synthesis, stem cell

## Abstract

Adult stem cells must limit their rate of protein synthesis, but the underlying mechanisms remain largely unexplored. Differences in protein synthesis among hematopoietic stem cells (HSCs) and progenitor cells did not correlate with differences in proteasome activity, total RNA content, mRNA content, or cell division rate. However, adult HSCs had more hypophosphorylated eukaryotic initiation factor 4E-binding protein 1 (4E-BP1) and 4E-BP2 as compared with most other hematopoietic progenitors. Deficiency for *4E-BP1* and *4E-BP2* significantly increased global protein synthesis in HSCs, but not in other hematopoietic progenitors, and impaired their reconstituting activity, identifying a mechanism that promotes HSC maintenance by attenuating protein synthesis.

## Abstract (precis)

Here, Signer et al. investigated the mechanism underlying the limited rate of protein synthesis in hematopoietic stem cells (HSCs). The authors found that adult HSCs had more hypophosphorylated eukaryotic initiation factor 4E-binding protein1 (4E-BP1) and 4E-BP2 as compared with most other hematopoietic progenitors, providing new insight into the mechanism by which HSCs attenuate protein synthesis.

Adult hematopoietic stem cell (HSC) maintenance depends on these cells making less protein per hour as compared with other hematopoietic progenitors (Signer et al. 2014). This does not reflect merely HSC quiescence, as dividing HSCs also make less protein per hour as compared with other hematopoietic progenitors. Several other adult stem cells also exhibit lower rates of protein synthesis as compared with progenitors in the same tissues, including neural stem cells (Llorens-Bobadilla et al. 2015), skeletal muscle stem cells (Zismanov et al. 2016), hair follicle stem cells (Blanco et al. 2016), and *Drosophila* germline stem cells (Sanchez et al. 2016). Like HSCs, each of these stem cells is depleted by genetic changes that increase their rate of protein synthesis, suggesting that this is a widely shared property of adult stem cells. However, there is limited insight into the mechanisms that suppress protein synthesis in stem cells.

Ribosome biogenesis limits protein synthesis in some stem cells. Quiescent neural stem cells express lower levels of ribosomal subunits and synthesize protein at a lower rate as compared with activated neural stem cells (Llorens-Bobadilla et al. 2015). Increased ribosome biogenesis in *Drosophila* germline stem cells increases protein synthesis, promoting differentiation and the loss of stem cells (Sanchez et al. 2016). Replication stress in HSCs from old mice transcriptionally silences ribosome genes, reducing ribosome biogenesis and perhaps impairing HSC function during aging (Flach et al. 2014). *Runx1*-deficient HSCs have impaired ribosome biogenesis, lower protein synthesis, and altered function (Cai et al. 2015). Defects in ribosome biogenesis impair HSC function (Le Bouteiller et al. 2013).

Phosphorylated eukaryotic initiation factor 2α (eIF2α) inhibits translation initiation and is part of the mechanism that limits protein synthesis in some stem cells. eIF2α can be phosphorylated under steady-state circumstances, although phosphorylation is increased by a wide range of stresses, including unfolded protein responses (Wek et al. 2006). Protein synthesis in skeletal muscle stem cells is limited by high levels of phosphorylated eIF2α, which promotes quiescence and stem cell maintenance (Zismanov et al. 2016). However, eIF2α function may vary among stem cells, as HSCs express relatively low levels of phosphorylated eIF2α (Signer et al. 2014), and activation of the unfolded protein response can be deleterious to individual HSCs (van Galen et al. 2014).

mTORC1 signaling promotes protein synthesis through phosphorylation and activation of ribosomal protein S6 kinase 1 (Brown et al. 1995) and phosphorylation and inhibition of eIF4E-binding proteins (4E-BPs) (Beretta et al. 1996; Brunn et al. 1997). Three genes (*Eif4ebp1*, *Eif4ebp2*, and *Eif4ebp3*) encode 4E-BPs that negatively regulate translation by binding the cap-binding protein eIF4E, inhibiting eIF4G binding, and preventing eIF4F complex assembly (eIF4E–eIF4G–eIF4A). Phosphorylation of 4E-BPs by mTORC1 weakens their binding to eIF4E, promoting eIF4F assembly and cap-dependent translation (Pause et al. 1994; Gingras et al. 1999).

4E-BP levels vary among tissues. Hematopoietic cells express abundant 4E-BP1 and 4E-BP2 but not 4E-BP3 (Tsukiyama-Kohara et al. 2001). 4E-BP-mediated translation inhibition does not generally affect global translation rates but particularly impairs the translation of certain subsets of mRNAs (Morita et al. 2013; Tahmasebi et al. 2014). Conditional *Pten* deletion increases mTORC1 signaling in HSCs (Lee et al. 2010), increasing 4E-BP phosphorylation (Magee et al. 2012), elevating protein synthesis, and promoting HSC depletion (Yilmaz et al. 2006; Signer et al. 2014). It is not clear whether 4E-BPs regulate HSC function, although knockdown of 4E-BP2 (Hartman et al. 2013) or eIF4E1 (Yang et al. 2014) in neural progenitors increases protein synthesis and promotes premature neuronal differentiation.

The synthesis of O-propargyl-puromycin (OP-Puro) has facilitated the quantification of protein synthesis in individual cells in vivo (Liu et al. 2012). OP-Puro enters the acceptor site of ribosomes and is incorporated into nascent polypeptide chains. The amount of protein synthesis per hour in individual cells in vivo can then be quantified based on OP-Puro incorporation (Liu et al. 2012; Signer et al. 2014). In this study, we examined potential mechanisms that might influence differences in protein synthesis between HSCs and restricted hematopoietic progenitors in adult mouse bone marrow.

## Results and Discussion

### HSCs do not have high proteasome activity

**Figure 1.**
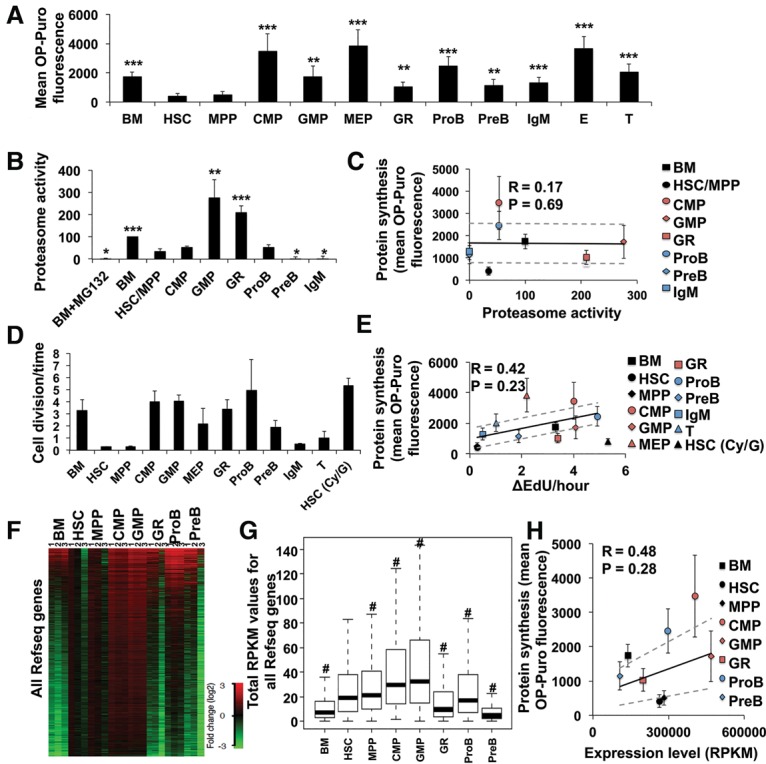
Protein synthesis rates in HSCs and progenitor cells show little correlation with proteasome activity, frequency of cell division, or RNA content. (*A*) OP-Puro incorporation by HSCs and progenitor cells in vivo. *n* = 8 mice in eight experiments. (*B*) Proteasome activity in HSCs and progenitor cells normalized to unfractionated bone marrow cells. *n* = 3 experiments. (*C*) Mean protein synthesis rate (from *A*) plotted against relative proteasome activity (from *B*). (*D*) The frequency of cell division for each population based on the rate of 5-ethynyl-2′-deoxyuridine (EdU) incorporation in vivo (see Supplemental Fig. S1B–M for primary data). *n* = 5 mice in two experiments. (*E*) Mean protein synthesis (from *A*) plotted against the rate of cell division (from *D*). (*F*) Gene expression values for all mouse RefSeq genes in each cell population (in total reads per kilobase of exonic length per million mapped reads [RPKM]). Log_2_ fold change of gene expression in each sample is shown relative to the average values in HSCs. (*G*) Total RPKMs of all mouse RefSeq genes in each cell population. Two-sided paired Wilcoxon rank-sum test was used for pair-wise comparisons between HSCs and each cell type. (#) *P* < 2.2 × 10^−16^. (*H*) Mean protein synthesis (from *A*) plotted against median total RPKMs (from *G*). Data represent mean ± SD unless indicated otherwise. The statistical significance of differences relative to HSCs in *A*, *B*, and *D* were assessed using a repeated-measures one-way analysis of variance (ANOVA) followed by Dunnett's test for multiple comparisons. (*) *P* < 0.05; (**) *P* < 0.01; (***) *P* < 0.001. Regression analyses in *C*, *E*, and *F* were performed excluding HSCs, which were plotted independently. Ninety-five percent confidence intervals (dashed lines) and Pearson's correlation coefficients (*R*) are shown.

We administered OP-Puro to mice, killed them 1 h later, and compared OP-Puro incorporation by subpopulations of bone marrow cells by flow cytometry. Consistent with our prior study (Signer et al. 2014), we observed significantly less OP-Puro incorporation into CD150^+^CD48^−^Lineage^−^ Sca-1^+^c-kit^+^ (CD150^+^CD48^−^LSK) HSCs (Kiel et al. 2005) and CD150^−^CD48^−^LSK multipotent progenitor cells (MPPs) (Oguro et al. 2013) as compared with restricted hematopoietic progenitors (Fig. 1A).

To test whether HSCs have high proteasome activity like embryonic stem cells (Vilchez et al. 2013), we compared the proteasome activity of HSCs versus restricted progenitors. We sorted 30,000 unfractionated bone marrow cells, CD48^−^LSK cells (HSCs/MPPs), CD34^+^CD16/32^low^CD127^−^Sca-1^−^LK common myeloid progenitors (CMPs) (Akashi et al. 2000), CD34^+^CD16/32^high^CD127^−^ Sca-1^−^LK granulocyte macrophage progenitors (GMPs) (Akashi et al. 2000), Gr-1^+^ myeloid, IgM^−^CD43^+^B220^+^ pro-B, IgM^−^CD43^−^B220^+^ pre-B (Hardy et al. 1991), and B220^+^IgM^+^ B cells. Proteasome activity in HSCs/MPPs was similar to CMPs and pro-B cells but significantly lower than unfractionated bone marrow cells, GMPs, and Gr-1^+^ cells (Fig. 1B; Supplemental Fig. S1A). Overall, proteasome activity showed little correlation with OP-Puro incorporation (*R* = 0.17, *P* = 0.69) (Fig. 1C).

### Little correlation between protein synthesis and cell division or RNA content

We administered the thymidine analog 5-ethynyl-2′-deoxyuridine (EdU) to mice and measured its incorporation by HSCs and restricted progenitors in the bone marrow after 2, 6, and 12–24 h to determine the frequency of cell division in each population (Fig. 1D). We observed a reasonably linear increase in the fraction of EdU^+^ cells over time in each population (Supplemental Fig. S1B–M). We observed a modest correlation between protein synthesis rates and cell division (*R* = 0.42, *P* = 0.23) (Fig. 1E). However, when we drove HSCs into cycle by treating them with cyclophosphamide and GCSF, we observed only a limited increase in protein synthesis (Fig. 1E). Therefore, the rate of protein synthesis in HSCs was not determined primarily by the rate at which they divide.

To test whether protein synthesis rates reflected total RNA content (largely ribosomal RNA), we quantified total RNA by nanofluidic electrophoresis using a BioAnalyzer. HSCs had amounts of total RNA similar to those of CMPs and pro-B cells and significantly more total RNA per cell than Gr-1^+^, pre-B, and unfractionated bone marrow cells (Supplemental Fig. S1N), each of which had higher protein synthesis rates than HSCs (Fig. 1A). Therefore, we observed little correlation between protein synthesis rates and total RNA content per cell (*R* = 0.32, *P* = 0.44) (Supplemental Fig. S1O).

We quantified the amount of mRNA per cell by RNA sequencing (RNA-seq) after adding RNA standards that allow normalization of transcript numbers to cell number (Fig. 1F; Loven et al. 2012). On average, HSCs had significantly more mRNA per cell as compared with pre-B cells, unfractionated bone marrow, and Gr-1^+^ cells but less mRNA per cell than CMPs, GMPs, and pro-B cells (Fig. 1G), all of which had higher protein synthesis rates than HSCs (Fig. 1A). Therefore, we observed little correlation between protein synthesis rates and mRNA content in hematopoietic cells (*R* = 0.48, *P* = 0.28) (Fig. 1H).

### HSCs have relatively high levels of hypophosphorylated 4E-BPs

**Figure 2.**
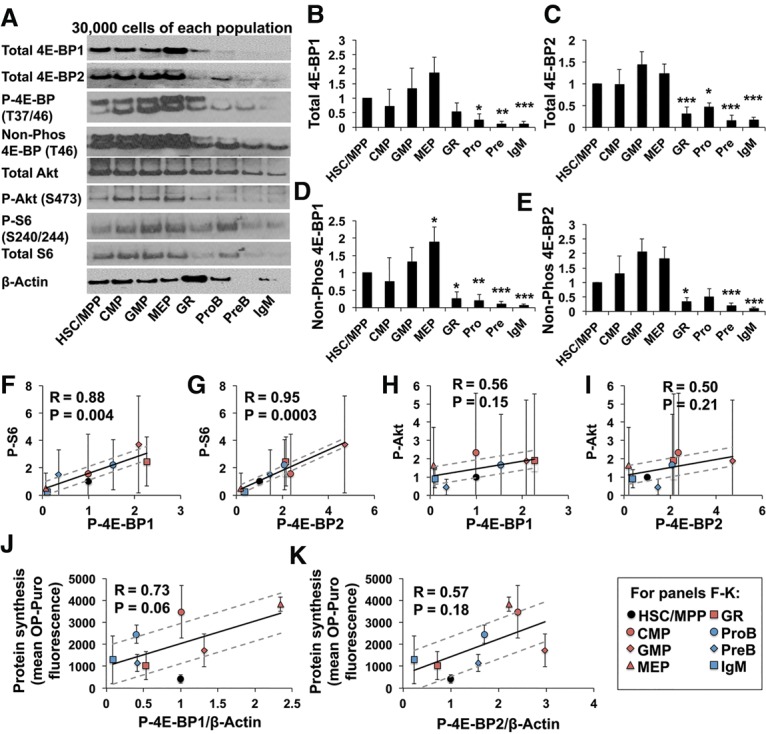
HSCs tend to have high levels of hypophosphorylated 4E-BPs relative to most other hematopoietic progenitors. (*A*) Western blot of 3 × 10^4^ cells from each cell population (one of four representative blots). Band intensity was normalized by cell number for each population. For phosphorylated and nonphosphorylated 4E-BP, the upper band is 4E-BP1, and the lower band is 4E-BP2 (see Supplemental Fig. S2A,B). (*B*–*E*) Quantification of band intensity for total 4E-BP1 (*B*), total 4E-BP2 (*C*), nonphosphorylated 4E-BP1 (*D*), and nonphosphorylated 4E-BP2 (*E*). (*F*,*G*) Mean intensity of phosphorylated S6 plotted against phosphorylated 4E-BP1 (*F*) or phosphorylated 4E-BP2 (*G*) levels for each cell population. (*H*,*I*) Mean intensity of phosphorylated Akt plotted against phosphorylated 4E-BP1 (*H*) or phosphorylated 4E-BP2 (*I*). (*J*,*K*) Mean protein synthesis (from Fig. 1A) plotted against phosphorylated 4E-BP1 (*J*) or 4E-BP2 (*K*) levels normalized to β-Actin. The regressions exclude HSCs/MPPs, which were plotted independently. Data in *B*–*K* represent mean ± SD from four experiments, including the one in *A*. The statistical significance of differences between HSCs/MPPs and other cell populations in *B*–*E* were assessed with one-way ANOVAs followed by Dunnett's test for multiple comparisons. (*) *P* < 0.05; (**) *P* < 0.01; (***) *P* < 0.001. For *F*–*K*, Pearson's correlation coefficient (*R*) and 95% confidence intervals (dashed lines) are shown.

We assessed the levels of total, phosphorylated, and nonphosphorylated 4E-BP1 and 4E-BP2 in 30,000 sorted cells from each population (Fig. 2A). Control experiments using peripheral blood from *4E-BP1*^−/−^ (Tsukiyama-Kohara et al. 2001), *4E-BP2*^−/−^ (Banko et al. 2005), and *4E-BP1*^−/−^*; 4E-BP2*^−/−^ mice (Le Bacquer et al. 2007) showed that the upper band was 4E-BP1, and the lower band was 4E-BP2 (Supplemental Fig. S2A,B). HSCs/MPPs expressed relatively high levels of total 4E-BP1 and 4E-BP2 as compared with most hematopoietic progenitors—significantly higher than Gr-1^+^, pro-B, pre-B, and IgM^+^ B cells (Fig. 2A–C). Levels of nonphosphorylated 4E-BP1 and 4E-BP2 were also significantly higher in HSCs/MPPs as compared with Gr-1^+^, pro-B, pre-B, and IgM^+^ B cells (Fig. 2D,E). The ratio of phosphorylated to total 4E-BP1/2 tended to be lower in HSCs as compared with restricted progenitors (Supplemental Fig. S2C,D). Differences in β-actin levels among cell populations represented differences in the amount of β-actin per cell, as lysates from equal numbers of cells were used in these experiments (Fig. 2A). Nonetheless, even when levels of total and nonphosphorylated 4E-BP1 and 4E-BP2 were normalized to differences in β-actin, they tended to remain higher in HSCs/MPPs as compared with Gr-1^+^, pro-B, pre-B, and IgM^+^ B cells (although not all of the differences were statistically significant) (Supplemental Fig. S2E–H).

Levels of phosphorylated S6 strongly correlated with levels of phosphorylated 4E-BP1 (*R* = 0.88, *P* = 0.004) (Fig. 2F) or phosphorylated 4E-BP2 (*R* = 0.95, *P* = 0.0003) (Fig. 2G). However, correlations between phosphorylated Akt and phosphorylated 4E-BP1 (*R* = 0.56, *P* = 0.15) (Fig. 2H) or phosphorylated 4E-BP2 (*R* = 0.50, *P* = 0.21) (Fig. 2I) were weaker. These data suggest that differences in 4E-BP phosphorylation among HSCs and progenitor cells likely reflect differences in mTORC1 signaling. Levels of phosphorylated 4E-BP1 (*R* = 0.73, *P* = 0.06) (Fig. 2J) and phosphorylated 4E-BP2 (*R* = 0.57, *P* = 0.18) (Fig. 2K) normalized to β-actin exhibited stronger correlations with protein synthesis rates than any other parameter that we tested. Levels of phosphorylated 4E-BP1 (*R* = 0.40, *P* = 0.37) (Supplemental Fig. S2I) and phosphorylated 4E-BP2 (*R* = 0.24, *P* = 0.6) (Supplemental Fig. S2J) that were normalized based on cell numbers did not exhibit as strong a correlation with protein synthesis.

**Figure 3.**
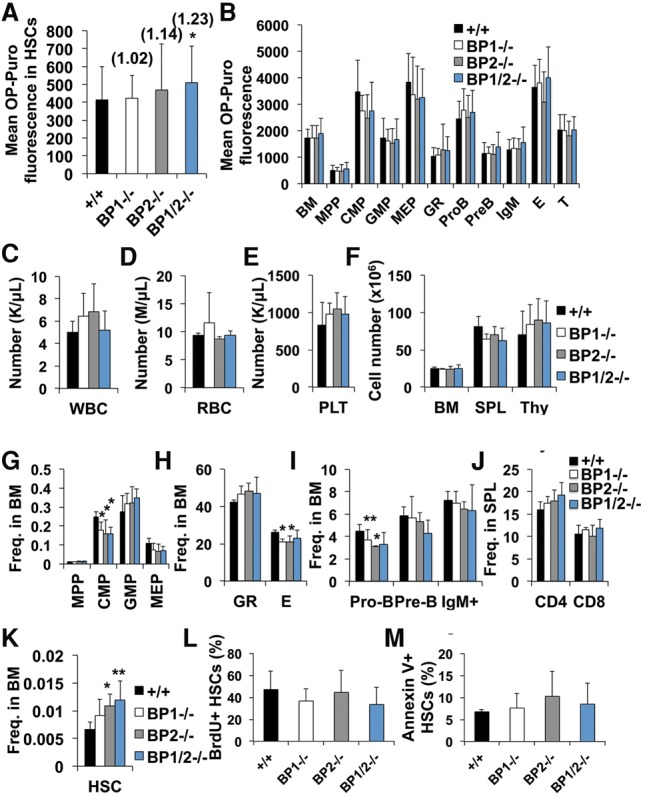
*4E-BP1/2*-deficient mice have HSCs with increased protein synthesis but largely normal hematopoiesis. (*A*) Mean OP-Puro incorporation by HSCs from *4E-BP1*^−/−^, *4E-BP2*^−/−^, *4E-BP1*^−/−^*;4E-BP2*^−/−^, or wild-type mice. *n* = 9 mice per genotype in nine experiments. The value relative to wild type is shown *above* each bar. (*B*) Mean OP-Puro incorporation into each restricted progenitor population from the same mice as in *A*. (*C*–*E*) Number of white blood cells (WBC), red blood cells (RBC), and platelets (PLT) in the peripheral blood. (*F*) Cell number in the bone marrow (BM; one femur and one tibia), spleen (SPL), and thymus (Thy). (*G*–*I*) Frequencies of MPPs, CMPs, GMPs, and megakaryocyte erythroid-restricted progenitors (MEPs) (*G*); Gr-1^+^ myeloid and Ter119^+^ erythroid cells (*H*); and pro-B, pre-B, and IgM^+^ B cells (*I*) in the bone marrow. (*J*) Frequencies of CD4^+^ and CD8^+^ T cells in the spleen. (*K*) Frequency of HSCs in the bone marrow. *n* = 6 mice per genotype in three experiments in *C*–*K*. (*L*) Frequency of BrdU^+^ HSCs after a 72-h pulse of BrdU in vivo. (*M*) Frequency of annexin V^+^ HSCs. *n* = 5–6 mice per genotype in four experiments in *L* and *M*. All data represent mean ± SD. The statistical significance of differences relative to wild type was assessed using one-way ANOVAs (repeated-measures in *A* and *B*) followed by Dunnett's test for multiple comparisons. (*) *P* < 0.05; (**) *P* < 0.01; (***) *P* < 0.001.

To test whether 4E-BPs limit the rate of protein synthesis in HSCs, we examined OP-Puro incorporation by HSCs and progenitors in *4E-BP1*^−/−^, *4E-BP2*^−/−^, and *4E-BP1*^−/−^*; 4E-BP2*^−/−^ mice. *4E-BP1*^−/−^ or *4E-BP2*^−/−^ HSCs did not exhibit significant changes in OP-Puro incorporation compared with wild-type HSCs (Fig. 3A). However, *4E-BP1*^−/−^*; 4E-BP2*^−/−^ HSCs exhibited a significant increase in protein synthesis as compared with wild-type HSCs (23%, *P* < 0.05) (Fig. 3A). The magnitude of this increase was close to that observed after *Pten* deletion, which leads to HSC depletion (Signer et al. 2014). We did not detect a statistically significant effect of *4E-BP1* deficiency, *4E-*BP2 deficiency, or combined *4E-BP1;4E-BP2* deficiency on the rate of protein synthesis in other hematopoietic progenitors (Fig. 3B). HSCs thus appear to be more sensitive to the effects of 4E-BP1/2 on global protein synthesis.

### *4E-BP1*^−/−^*;4E-BP2*^−/−^ HSCs have impaired regenerative activity

**Figure 4.**
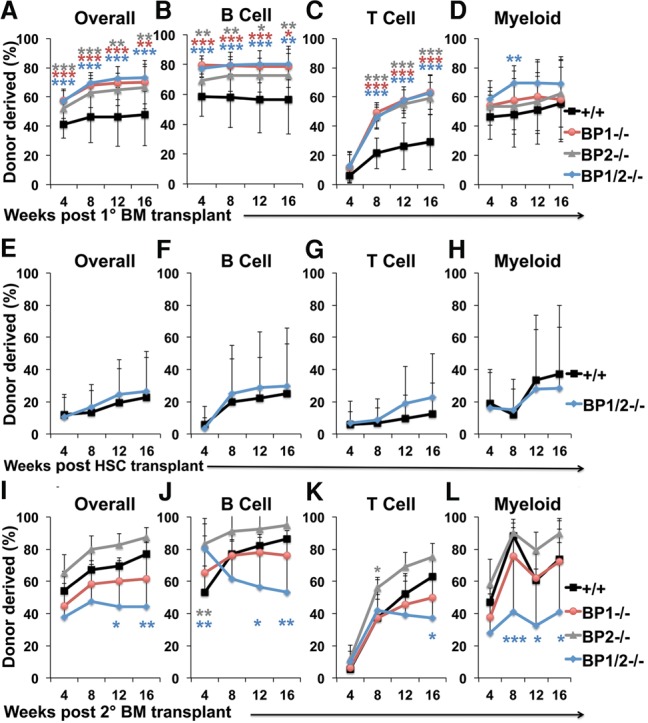
*4E-BP1*^−/−^*;4E-BP2*^−/−^ HSCs have impaired reconstituting potential upon serial transplantation. (*A*–*D*) Donor cell engraftment when 5 × 10^5^ bone marrow cells from mice of the indicated genotypes were transplanted along with 5 × 10^5^ recipient bone marrow cells into irradiated mice. Donor cell engraftment in the bone marrow is shown in Supplemental Figure S3A. (*E*–*H*) Donor cell engraftment when 10 HSCs from mice of the indicated genotypes were transplanted with 3 × 10^5^ recipient bone marrow cells into irradiated mice. (*I*–*L*) Donor cell engraftment after serial transplantation of 3 × 10^6^ bone marrow cells from primary recipients in *A*–*D* into secondary recipient mice. *n* = 8–11 recipients per genotype from two primary donors from wild type and three primary donors from each of the *4E-BP*-deficient genotypes. Data represent mean ± SD. The statistical significance of differences relative to wild-type were assessed with one-way ANOVAs followed by Dunnett's test for multiple comparisons in *A*–*D* and *I*–*L* and a two-tailed Student's *t*-test in *E*–*H*. (*) *P* < 0.05; (**) *P* < 0.01; (***) *P* < 0.001.

*4E-BP1*^−/−^, *4E-BP2*^−/−^, and *4E-BP1*^−/−^*;4E-BP2*^−/−^ compound mutant mice all exhibited relatively normal hematopoiesis (Fig. 3C–J). HSC frequency appeared to be higher in *4E-BP2*^−/−^ (65% ± 32%, *P* < 0.05) (Fig. 3K) and *4E-BP1*^−/−^*; 4E-BP2*^−/−^ (80% ± 53%, *P* < 0.01) mutant mice (Fig. 3K) as compared with controls. This modest increase in HSC frequency was not associated with significant changes in the frequency of dividing (Fig. 3L) or dying (Fig. 3M) HSCs. This suggests that the increase in HSC frequency may have been caused by a lower rate of differentiation by *4E-BP1/2*-deficient HSCs. We also observed modest but significant declines in the frequencies of CMPs, Ter119^+^ erythroid progenitors, and pro-B cells in the bone marrow of some *4E-BP*-deficient mice as compared with controls (Fig. 3G–I).

To test the effect of *4E-BP* deficiency on HSC function, we performed competitive reconstitution assays in irradiated mice. Bone marrow cells (5 × 10^5^ cells) from *4E-BP1*^−/−^, *4E-BP2*^−/−^, *4E-BP1*^−/−^*;4E-BP2*^−/−^, or wild-type mice (all CD45.2^+^) were transplanted with equal numbers of wild-type recipient bone marrow cells (CD45.1^+^) into irradiated mice (CD45.1^+^). Recipients of *4E-BP*-deficient bone marrow cells had significantly higher levels of donor-derived hematopoietic cells in their blood 4–16 wk after transplantation (Fig. 4A–D). To test whether this increased reconstitution reflected increased HSC frequency in the bone marrow of *4E-BP*-deficient donors (Fig. 3K) or increased function of *4E-BP*-deficient HSCs, we competitively transplanted 10 CD150^+^CD48^−^LSK HSCs from *4E-BP1*^−/−^*;4E-BP2*^−/−^ or wild-type donors into irradiated recipient mice along with recipient bone marrow cells. *4E-BP*-deficient HSCs gave normal levels of donor cell reconstitution (Fig. 4E–H). The higher level of donor cell reconstitution by *4E-BP*-deficient bone marrow cells thus reflects higher HSC frequency in the donor bone marrow rather than increased reconstituting activity by *4E-BP*-deficient HSCs.

Although *4E-BP2*^−/−^ and *4E-BP1*^−/−^*;4E-BP2*^−/−^ mutant mice had increased HSC frequency in their bone marrow as compared with controls (Fig. 3K), the frequency of donor HSCs in primary recipient mice after bone marrow transplantation did not significantly differ between recipients of *4E-BP*-deficient cells as compared with control cells (Supplemental Fig. S3B). These data raised the possibility that *4E-BP* deficiency may impair HSC self-renewal after transplantation.

To assess HSC self-renewal potential, we performed secondary transplants of 3 × 10^6^ bone marrow cells from primary recipient mice with levels of donor cell reconstitution nearest the median values in each treatment. Deficiency for only *4E-BP1* or *4E-BP2* did not significantly affect donor cell reconstitution in secondary recipient mice as compared with control cells. In contrast, secondary recipients of *4E-BP1*^−/−^*;4E-BP2*^−/−^ cells had significantly less donor cell reconstitution in all lineages 16 wk after transplantation as compared with secondary recipients of wild-type donor cells (Fig. 4I–L). These data suggest that *4E-BP1*^−/−^*;4E-BP2*^−/−^ HSCs have reduced self-renewal potential as compared with control HSCs.

4E-BP1 and 4E-BP2 likely regulate the translation of a subset of mRNAs in HSCs. Increased expression of these mRNAs likely increases HSC frequency (Fig. 3K) and reconstituting capacity in the bone marrow of *4E-BP1*/2^−/−^ mice (Fig. 4A–D) while reducing HSC self-renewal upon serial transplantation (Fig. 4I–L). Nonetheless, other mechanisms must also limit protein synthesis in HSCs, as protein synthesis rates in *4E-BP1/2*^−/−^ HSCs remained significantly lower than in *4E-BP1/2*^−/−^ restricted hematopoietic progenitors. Moreover, both GMPs and megakaryocyte erythroid-restricted progenitors (MEPs) had higher levels of hypophosphorylated 4E-BPs relative to HSCs (Fig. 2D,E) and yet still had higher levels of global protein synthesis (Fig. 1A), perhaps because global protein synthesis in these cells is not as sensitive to 4E-BP1/2 as in HSCs (Fig. 3B).

## Materials and methods

### Mice

*Eif4ebp1*^−/−^ (Tsukiyama-Kohara et al. 2001), *Eif4ebp2*^−/−^ (Banko et al. 2005), and *Eif4ebp1*^−/−^*;Eif4ebp2*^−/−^ (Le Bacquer et al. 2007) mice have been described previously. These mice were backcrossed for at least 10 generations onto a C57BL background. C57BL/Ka-Thy-1.1 (CD45.2) mice were used as wild type throughout this study. C57BL/Ka-Thy-1.2 (CD45.1) mice were used as transplant recipients. Male and female mice between 8 and 14 wk old were used in all studies. Cyclophosphamide and GCSF were administered as described (Signer et al. 2014). All mice were housed in the Animal Resource Center at the University of Texas Southwestern Medical Center, and all protocols were approved by the University of Texas Southwestern Medical Center Institutional Animal Care and Use Committee.

### Proteasome activity assay

Proteasome activity was determined by measuring the rate of hydrolysis of Suc–Leu–Leu–Val–Tyr–7-amino-4-methylcoumarin (AMC). This substrate is specific for the proteasome under the assay conditions used here and is hydrolyzed by all proteasome holoenzymes. The activity reported by this assay represents the total enzymatic capacity of the cellular proteasome and is typically dominated by the 26S proteasome (Sasaki et al. 1984; Demartino and Gillette 2007). From each population, 3 × 10^4^ cells were double-sorted into 96-well black plates. One-hundred microliters of buffer containing 20 mM Tris-HCl (pH 7.6), 1 mM 2-mercaptoethanol, 5 mM MgCl_2_, 1 mM ATP, and 0.4% NP-40 was added to each well followed immediately by 50 µL of 200 µM Suc–Leu–Leu–Val–Tyr–AMC (Bachem). The fluorescence of free AMC generated by proteasomal hydrolysis of the substrate was monitored continuously (one read per minute for 180 min at 37°C) at 360^ex^/460^em^ using a BioTek Synergy II plate reader. Controls included reactions conducted in the absence of cells, in the presence of 10 mM MG132 (UBPBio), or using purified bovine 26S proteasome instead of cells. Data are expressed as arbitrary fluorescent units per well.

### The rate of cell division

Two milligrams of EdU (Thermo Scientific) in PBS was injected intraperitoneally per mouse every 6 h. Mice were analyzed 2, 6, 12, and 24 h after EdU administration. These short labeling times minimized the extent to which results were affected by the entry of labeled cells into each population through the maturation of upstream progenitors, the exit of labeled cells through differentiation into downstream progeny, or saturation of labels in rapidly dividing cell populations. Bone marrow cells (3 × 10^6^ cells) were stained with antibodies as described in the Supplemental Material. Cells were fixed and permeabilized, and the azide–alkyne cycloaddition was performed as with OP-Puro detection and analyzed by flow cytometry. For cyclophosphamide- and GCSF-treated mice, BrdU was administered in place of EdU and was detected with the BrdU flow kit.

## Supplementary Material

Supplemental Material
