# Supplementary material for: The rate of protein synthesis in hematopoietic stem cells is limited partly by 4E-BPs
Source: Genes Dev. 2016 Aug 1;30(15):1698–703. doi: 10.1101/gad.282756.116 (PMC5002975; doi:10.1101/gad.282756.116)
Supplement: Supplemental Material [file supp_30_15_1698__index.html]

The rate of protein synthesis in hematopoietic stem cells is limited partly by 4E-BPs — Supplemental Material 

# The rate of protein synthesis in hematopoietic stem cells is limited partly by 4E-BPs

## Supplemental Material

**Files in this Data Supplement:**

- Supplemental\_Material.pdf
