# Supplementary material for: The rate of protein synthesis in hematopoietic stem cells is limited partly by 4E-BPs
Source: Genes Dev. 2016 Aug 1;30(15):1698–703. doi: 10.1101/gad.282756.116 (PMC5002975; doi:10.1101/gad.282756.116)
Supplement: Supplemental Material [file supp_gad.282756.116_Supplemental_Material.pdf]

Supplemental Material for

The rate of protein synthesis in hematopoietic stem cells is limited partly by 4E-BPs

Robert A.J. Signer[1,3*], Le Qi[1], Zhiyu Zhao[1], David Thompson[2], Alla A. Sigova[4], Zi Peng Fan[4], George N. DeMartino[2], Richard A. Young[4,5], Nahum Sonenberg[6], and Sean J. Morrison[1*]

[1]Howard Hughes Medical Institute, Children's Research Institute, Department of Pediatrics,

[2]Department of Physiology, University of Texas Southwestern Medical Center, Dallas, TX, 75390, USA

[3]Division of Regenerative Medicine, Department of Medicine, Moores Cancer Center, University of California, San Diego, La Jolla, CA, 92093, USA

[4]Whitehead Institute for Biomedical Research, [5]Department of Biology, Massachusetts Institute of Technology, Cambridge, MA, USA

[6]Department of Biochemistry and Goodman Cancer Centre, McGill University, Montreal, Quebec, H3A 1A3, Canada

*Corresponding authors:

rsigner@ucsd.edu

Sean.Morrison@UTSouthwestern.edu

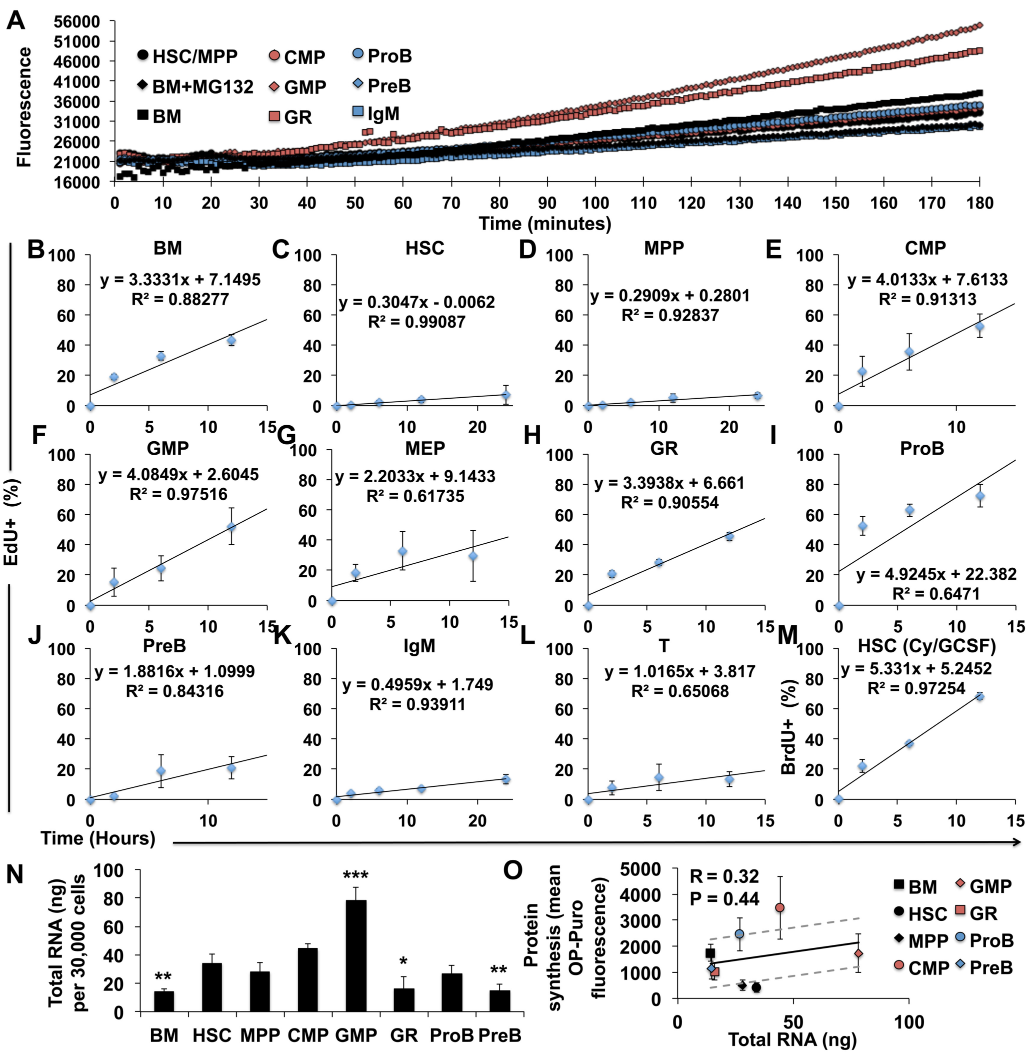

Supplemental Figure S1. Differences in proteasome activity, frequency of cell division, and total RNA content among hematopoietic stem and progenitor cell populations. (A) Representative plots of fluorescence over time in the proteasome activity assay. Addition of MG132 was added to unfractionated bone marrow cells as a negative control. Data are expressed as arbitrary fluorescence units. (B-L) Scatter plots show the frequency of EdU^+ cells within each population at various times after EdU administration in vivo. EdU incorporation was assessed in each population by flow cytometry 2, 6, 12, and 24 (HSC, MPP and IgM^+ only) hours after administation and in an untreated control group (0 hours) (n=5 mice/time point in 2 independent experiments). The equations describe the change in $\%EdU^+$ cells per hour. (M) Mice were treated with cyclophosphamide followed by two daily injections of G-CSF before BrdU administration to drive HSCs into cycle (n=3 mice/time point). (N) Total RNA levels from $3x10^4$ cells from each cell population (pooled cells 6-7 mice in each of 3 independent experiments). (O) Scatter plot of mean OP-Puro incorporation (from Fig. 1A) plotted against total RNA content (from N). Regression analysis was performed and 95% confidence intervals were determined (dashed lines). The regression analysis was performed excluding HSCs, which were plotted independently. Pearson's correlation coefficient R is shown. All data represent mean ± s.d. The statistical significance of differences relative to HSCs in panel N were assessed with a one-way ANOVA followed by Dunnett's test for multiple comparisons. *P<0.05, **P<0.01, ***P<0.001.

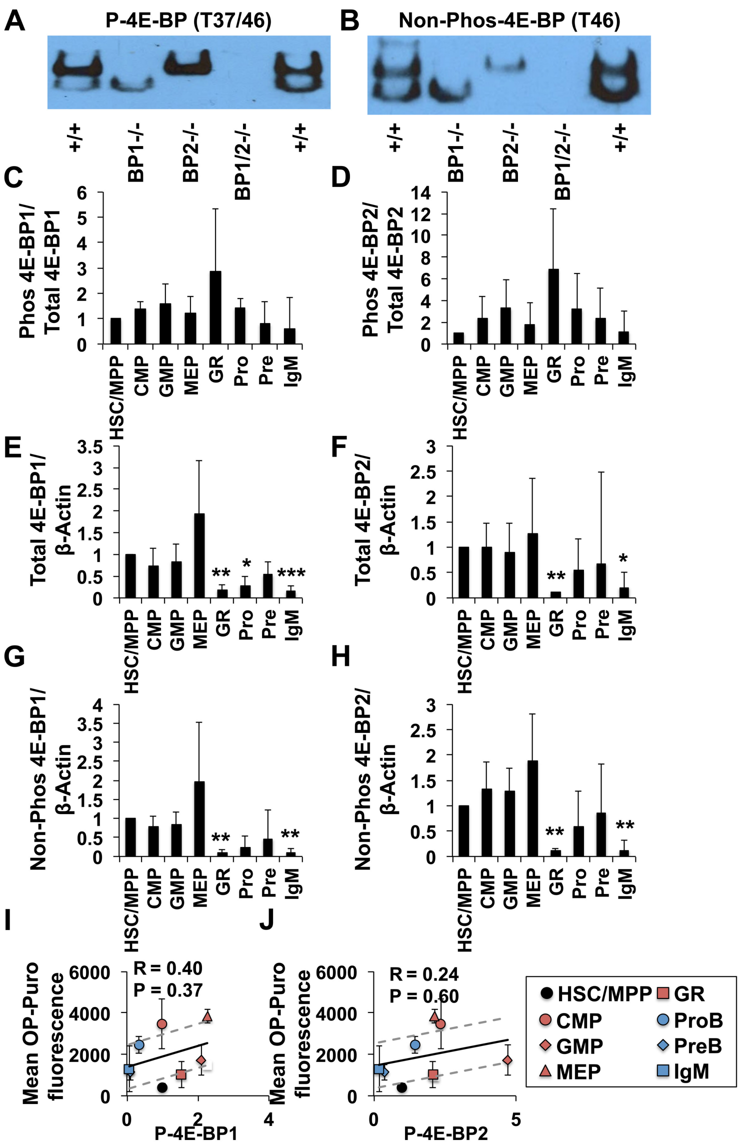

Supplemental Figure S2. Western analysis of phosphorylated and non-phosphorylated 4E-BP levels. (A) Western blot analysis of peripheral blood cells from mice of the indicated genotypes indicating that the top band is phosphorylated 4E-BP1 and the bottom band is phosphorylated 4E-BP2. (B) Western blot analysis of peripheral blood cells from mice of the indicated genotypes indicating that the top band is non-phosphorylated-4E-BP1 and the bottom band is non-phosphorylated-4E-BP2. (C) Ratio of phosphorylated 4E-BP1 to total 4E-BP1 in the indicated cell populations. (D) Ratio of phosphorylated 4E-BP2 to total 4E-BP2. (E-H) Total 4E-BP1 (E) total 4E-BP2 (F), non-phosphorylated 4E-BP1 (G), and non-phosphorylated 4E-BP2 (H) in each hematopoietic stem or progenitor cell population normalized to β-Actin levels based on quantification of band intensities. (I-J) Mean protein synthesis (from Fig. 1A) plotted against phosphorylated 4E-BP1 (I) or 4E-BP2 (J) levels. The regressions excluded HSCs/MPPs, which were plotted independently. Pearson's correlation coefficient R and 95% confidence intervals (dashed lines) are shown. Data in C-H represent mean ± s.d. from four independent experiments including the one shown in Fig. 2A. The statistical significance of differences relative to HSCs/MPPs were assessed using one-way ANOVAs followed by Dunnett's test for multiple comparisons in panels C-H. *P<0.05, **P<0.01, ***P<0.001.

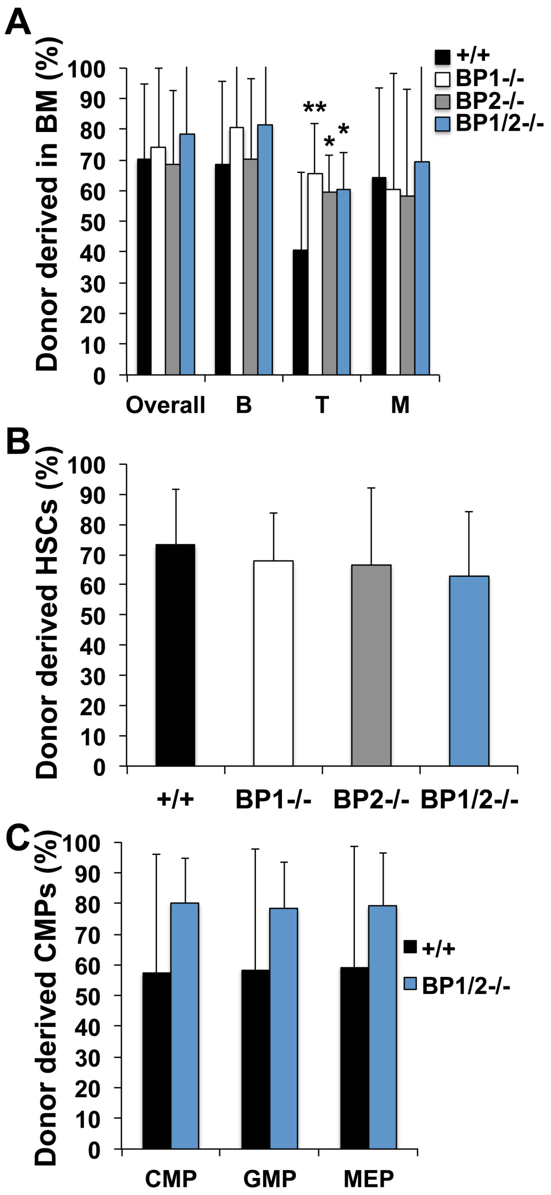

Supplemental Figure S3. Reconstitution of the bone marrow by *4E-BP*-deficient cells after transplant. (A) Donor-derived hematopoietic cells, B cells, T cells and myeloid cells in the bone marrow (femur and tibia) of recipient mice shown in Fig. 4A-D 16 weeks after transplantation (n=14 or 15 total recipients per genotype from 3 independent experiments). (B) Donor-derived HSC frequency in the bone marrow (femur and tibia) of primary recipient mice shown in A-D at 16 weeks after transplantation (n=14-15 recipients/genotype in 3 experiments). (C) Donor derived CMPs, GMPs and MEPs in the bone marrow (femur and tibia) of reconstituted mice 9 months after transplantation (n=6-9 recipients per genotype). Data represent mean ± s.d. The statistical significance of differences relative to wild-type were assessed with a one-way ANOVA followed by Dunnett's test for multiple comparisons in A-B and a two-tailed Student's t-test in C. *P<0.05, **P<0.01.

SUPPLEMENTAL MATERIALS AND METHODS

Measuring protein synthesis

The rate of protein synthesis was measured as described previously (Signer et al. 2014). O-propargyl-puromycin (OP-Puro; 50 mg/kg body mass; pH 6.4-6.6 in PBS) was injected intraperitoneally and mice were euthanized 1 hour later. $3x10^6$ bone marrow cells were stained with antibodies against cell surfaces markers as described below. After washing, cells were fixed, permeabilized, and the azide-alkyne cycloaddition was performed using the Click-iT Cell Reaction Buffer Kit (Life Technologies) with 5 µM azide conjugated to Alexa Fluor 555 (Life Technologies).

Flow cytometry and cell isolation

Bone marrow, spleen, and thymus cells were isolated and harvested as described (Signer et al. 2014). For flow cytometric analysis and isolation of hematopoietic cells, cells were incubated with combinations of antibodies to the following cell-surface markers conjugated to FITC, PE, PerCP-Cy5.5, APC, PE-Cy7, eFluor 660, Alexa Fluor 700, APC-eFluor 780, or biotin: CD3ε (17A2), CD4 (GK1.5), CD5 (53-7.3), CD8α (53-6.7), CD11b (M1/70), CD16/32 (FcYRII/III; 93), CD24 (M1/69), CD25 (PC61.5), CD34 (RAM34), CD43 (1B11), CD44 (IM7), CD45.1 (A20), CD45.2 (104), CD45R (B220; RA3-6B2), CD48 (HM48-1), CD71 (C2), CD117 (c-kit; 2B8), CD127 (IL7Rα; A7R34), CD138 (281-2), CD150 (TC15-12F12.2), Ter119 (TER-119), Sca1 (D7, E13-161.7), Gr-1 (RB6-8C5), and IgM (II/41). For HSCs and MPPs, Lineage markers included CD3, CD5, CD8, B220, Gr-1, and Ter119. For CMPs, GMPs, and MEPs, these Lineage markers were supplemented with CD4 and CD11b. Biotinylated antibodies were visualized by incubation with PE-Cy7 conjugated streptavidin. Antibodies were from BD Biosciences, eBiosciences, or BioLegend. Incubations were for approximately 30 minutes on ice. HSCs, MPPs, CMPs, GMPs, and MEPs were pre-enriched by selecting c-kit$^+$ cells using paramagnetic microbeads and an autoMACS magnetic separator (Miltenyi).

Dead cells were excluded using 4’,6-diamidino-2-phenylindole (DAPI). Apoptotic cells were identified using APC annexin V (BD Biosciences). Proliferation was assessed by administering 5-bromo-2’-deoxyuridine (BrdU; Sigma) to mice. BrdU dissolved in PBS was administered with an initial intraperitoneal injection (100 µg/g) and then mice were maintained on drinking water containing 1 mg/ml BrdU for 72 hours. BrdU incorporation in HSCs was measured using the APC BrdU Flow Kit (BD Biosciences). Data acquisition and cell sorting were performed on a FACSAria or FACSCanto (BD Biosciences). Fractions were double sorted to ensure high purity. Data were analyzed with FlowJo (Tree Star).

RNAseq analysis

RNA was extracted from $3x10^4$ cells from each cell population using the mirVana miRNA isolation kit (Thermo Scientific). Total RNA was quantified using a Bioanalyzer (Agilent). To assess mRNA content we performed RNAseq on the total RNA extracted from each cell population, adding equal amounts of 92 spiked-in RNA standards to each sample. Since the amount of spiked-in RNA standards added to each sample was known, the relationship between RPKM values and the number of transcripts for each spiked-in RNA could be determined by regression analysis (Loven et al. 2012). This relationship was used to calculate the total number of transcripts (total mRNA) in each sample. RNAseq reads were aligned using Bowtie software (Langmead et al. 2009) to NCBI build 37 (mm9) of the mouse genome with the settings: -e 70 -k 1 -m 2 - n 2. The RPKM for each RefSeq gene and synthetic spike-in RNA was calculated using RPKM_count.py (v2.3.5) counting only exonic reads (-e option). Loess regression from R affy package was used to renormalize the RPKM values by using only the spike-in RNA to fit the loess with default parameters. Only the spike-in RNAs whose abundance could be robustly quantified (RPKM values ≥ 1) were used in the loess normalization.

Transplantation

Recipient mice (CD45.1) were administered a minimum lethal dose of radiation using an XRAD 320 X-ray irradiator (Precision X-Ray Inc.) to deliver two doses of approximately 540 rad (1,080 rad in total) at least 3 hours apart. Cells were injected into the retro-orbital venous sinus of anesthetized recipients as described (Signer et al. 2014).

Western blot analysis

$3x10^4$ cells from each population were sorted into 10% trichloracetic acid (TCA, Sigma), incubated on ice for 15 minutes and centrifuged at 16,100 x g at 4°C for 15 minutes. Precipitates were washed in acetone twice and dried. Pellets were solubilized in 9M urea, 2% Triton X-100, and 1% DTT. LDS loading buffer (Life Technologies) was added and the pellet was heated at 70°C for 10 minutes. Samples were separated on Bis-Trispolyacrylamide gels (Life Technologies) and transferred to PVDF membrane (Millipore). Western blotting was performed according to protocols from Cell Signaling Technologies and blots were developed with the SuperSignal West Femto chemiluminescence kit (Thermo Scientific). Blots were stripped with 1% SDS, 25 mM glycine (pH 2) prior to reprobing. Primary antibodies (Cell Signaling Technologies): phos-4E-BP1 (T37/46; 236B4), 4E-BP1 (9452), 4E-BP2 (2845), non-phos-4E-BP1 (T46; 87D12), phos-Akt (Ser473; D9E), AKT (C67E7), phos-S6 (Ser240/244; polyclonal), S6 (5G10), and β-Actin (AC-74; Sigma). Band intensity was quantified with ImageJ.

SUPPLEMENTAL REFERENCES

Langmead B, Trapnell C, Pop M, Salzberg SL. 2009. Ultrafast and memory-efficient alignment of short DNA sequences to the human genome. *Genome Biol* **10**: R25.

Loven J, Orlando DA, Sigova AA, Lin CY, Rahl PB, Burge CB, Levens DL, Lee TI, Young RA. 2012. Revisiting global gene expression analysis. *Cell* **151**: 476-482.

Signer RA, Magee JA, Salic A, Morrison SJ. 2014. Haematopoietic stem cells require a highly regulated protein synthesis rate. *Nature* **509**: 49-54.
